# Cross-segment spinal plasma cell granuloma:a case report

**DOI:** 10.1186/s12891-020-03759-4

**Published:** 2020-11-12

**Authors:** Renqin Lin, Shenglin Wang, Jianhua Lin, Zhenzhen Zhang, Xuanwei Chen

**Affiliations:** 1 Department of Orthopedic Surgery, the First Affiliated Hospital of Fujian Medical University, Fuzhou, Fujian 350005 P.R. China; 2 Department of Pathology, the First Affiliated Hospital of Fujian Medical University, Fuzhou, Fujian 350005 P.R. China

**Keywords:** Plasma cell granuloma, Thoracolumbar spine, Surgery, Case report

## Abstract

**Background:**

Plasma cell granuloma (PCG) is a rare non-neoplastic entity, with the precise etiology remaining unclear. Vertebra-affected spinal PCG has not been reported yet. This report presented a case with cross-segment spinal PCG in thoracolumbar region.

**Case presentation:**

A 32-year-old male patient presented to the authors’ hospital since his health check-up results showed osteolytic lesions in the thoracolumbar spine. He felt asymptomatic throughout the course. Radiological examination revealed destructive changes at T12 and L1 vertebrae. Whereas laboratory examination excluded malignant tumor. The results of routine incisional biopsy remained inconclusive, thereby necessitating complete excision of the lesions. Finally, the infiltration of plasma cells observed by pathological examination of the surgical specimen confirmed the diagnosis of PCG.

**Conclusions:**

To the authors’ knowledge, this was the first case of cross-segment spinal PCG with osteolytic property. The possibility of PCG should be considered for the diagnosis and differential diagnosis of an osteolytic lesion in the spine. Since the etiology of PCG is unknown, the disorder was confirmed based on excluded diagnosis. Surgical resection is recommended both for the definite diagnosis and treatment of spinal PCG.

## Background

Plasma cell granuloma (PCG), firstly mentioned by Bahadori and Liebow, is a pseudotumor-like condition characterized by the polyclonal proliferation of plasma cells. The etiopathogenesis of PCG is still unknown. Although PCG is regarded as a generally benign and nonrecurring lesion, the outcome of those with local aggressiveness or recurrence may become complicated. PCG is commonly seen in lung and occasionally seen in esophagus, mouth, stomach, thyroid gland, intestine, kidney, lymph node, and skin. Additionally, PCG was ever found in jaw and temporal bone. However, vertebra involved PCG has not been reported yet. We here reported the first case of cross-segment PCG occurred in the thoracolumbar spine. This report was approved by the Ethics Committee of our hospital. Informed consent was obtained from the patient concerning the data submitted for publication.

## Case presentation

**Fig. 1:**
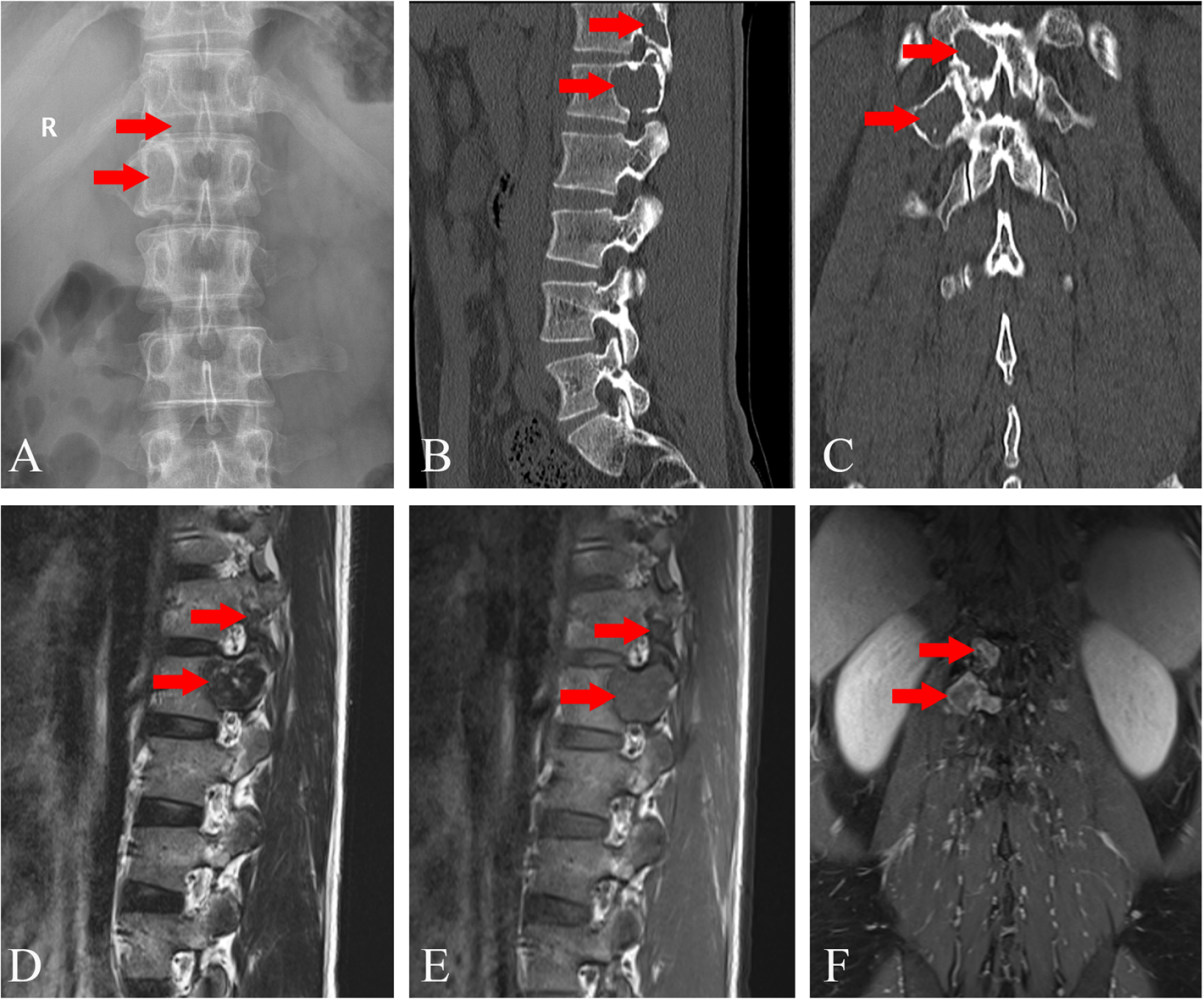
**a.** X-ray reveals lesions at T12 and L1 vertebrae; **b.** Sagittal and **c.** coronal CT scan reveals osteolytic lesions; The lesions are **d.** T1-weighted and **e.** T2-weighted iso- and hypo-intensities, with **f.** ring-enhancement of the peripheral area on MRI

**Fig. 2:**
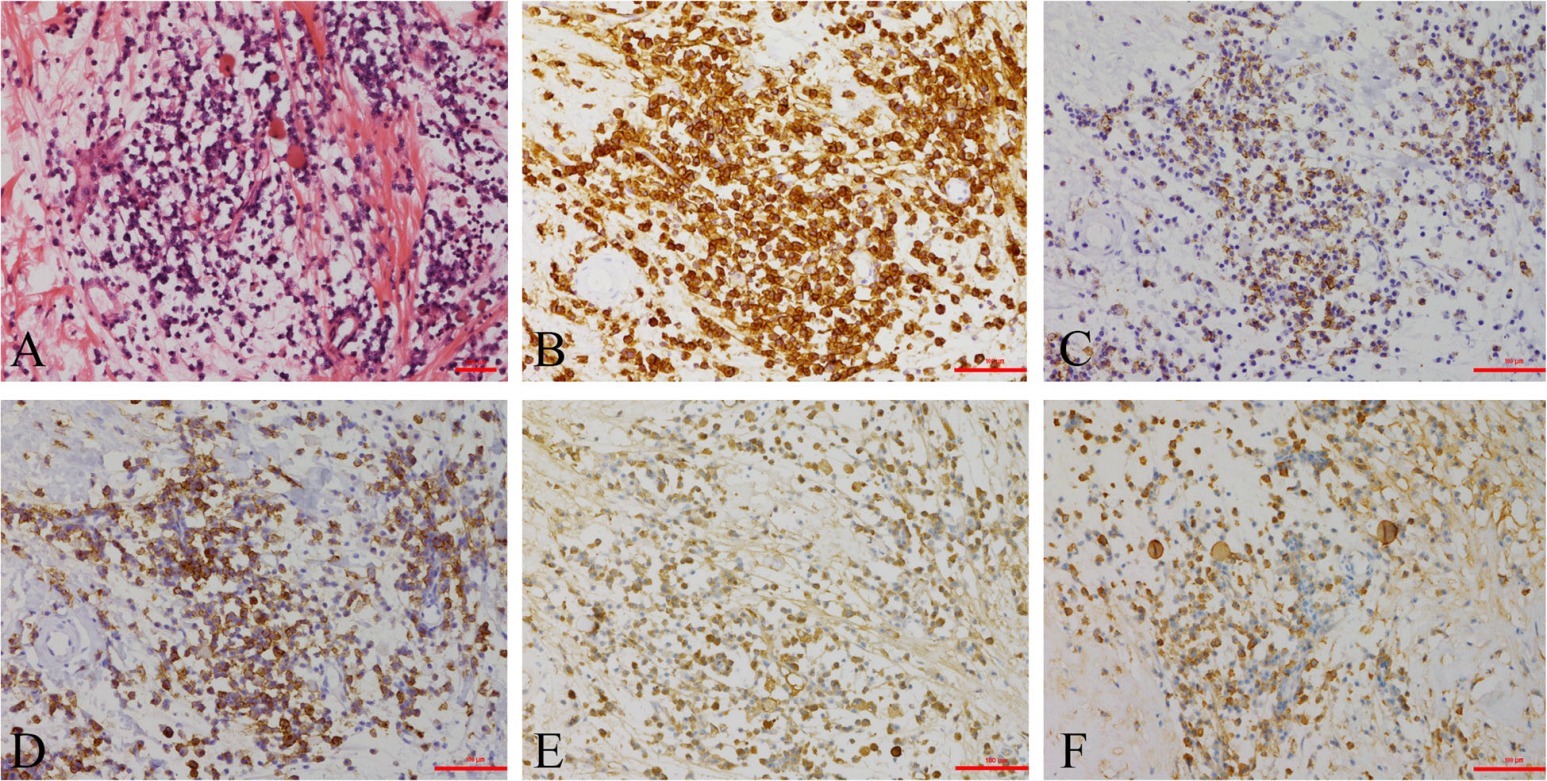
**a.** HE staining reveals plasma cells and Russell bodies. Immunohistochemical staining reveals strong expression of **b.** CD38,and mild expression of **c.** CD138, **d.** EMA, **e.** kappa and **f.** lambda light chains. Original magnification × 200

A 32-year-old man presented to the local hospital for health check-up and the computed tomography (CT) scan pitched upon osteolytic lesions in his thoracolumbar spine. Three days later, he was referred to our hospital for further management. Throughout the course, he had no pain or fever, with negative neuroradiological manifestation in his lower limbs. His past medical history was unremarkable. He had no history of keeping bird or travelling to the epidemic area in the past few years. Risk factors for tuberculosis and HIV exposure were not found. Physical examination showed no tenderness or obvious mass in the thoracolumbar level. No neurological dysfunction or lymphadenopathy was noted. The X-ray film observed a radiolucent lesion at T12 vertebra and enlargement of the right pedicle of L1 (Fig. 1a). The CT scan showed aggressive and destructive lesions at T12 and L1 vertebrae, with the facet joint not affected (Fig. 1b, c). The magnetic resonance (MR) image revealed lesions with T1-weighted heterogeneous iso- and hypo-intensities, and T2-weighted mixed signal of high and low intensities at T12 and L1 vertebrae (Fig. 1d, e). Contrast-enhanced T1-weighted MR image showed strong ring-enhancement of the peripheral lesion and low enhancement of the lesion body (Fig. 1f). Bone scintigraphy revealed no lesion with elevated radioactive signal in the whole skeleton. Routine laboratory examinations, including biochemistry and hemogram, were all within normal limits. The tumor marker chip test, galactomannan (GM) test, G-test for mycotic infection, immunoprotein electrophoresis for multiple myeloma (MM), T-SPOT test for tuberculosis, HIV and rapid plasma reagin (RPR) test showed all negative results. The lesions were even undiagnosed with routine incisional biopsy, thereby necessitating complete excision of the lesions for both diagnosis and treatment. Under general anesthesia in prone position, surgical resection involving posterior partial corpectomy of the T12 and L1 vertebrae was done. Intraoperative specimens were sent for pathological examination. Hematoxylin and eosin (HE) staining revealed infiltration of inflammatory cells, consisted of cells with eccentrically placed nuclei in cartwheel-shaped chromatin and lymphocytes. Eosinophil leukocytes and Russell bodies were also observed to form eosinophilic globules (Fig. 2a). The immunohistochemical analysis revealed cytoplasmic staining for CD38, CD138, epithelial membrane antigen (EMA), kappa and lambda light chains (Fig. 2b-f), CD163, CD68, CD19, and leukocyte common antigen (LCA). The maximum labeling index of Ki-67 was less than 1%. Postoperative period was uneventful. At two-year follow-up, the patient reported no residual symptom or sign of recurrence.

## Discussion and conclusions

PCG is an uncommon non-neoplastic lesion with the polyclonal proliferation of plasma cells [1]. This disorder is known to occur in numerous sites, with the lung mostly reported [2–6]. Despite the fact that the clinical features vary according to the localization of lesion, in most cases no significant symptom is observed [3]. Intraspinal PCG has been reported, complicated with progressive neurological deficit [7–12]. A sacral PCG case even manifested as paraneoplastic syndrome [11]. We here reported the first case of spinal PCG in thoracolumbar region with vertebra destructions at T12 and L1. Since there was no compression on spinal canal, the neurological dysfunction was negative. Laboratory and imaging examinations failed to make a definite diagnosis. Finally, surgical excision of the lesions followed by pathological examination revealed the infiltration of plasma cells without neoplastic properties. Immunohistochemical expressions of plasma cell-specific markers including CD38, CD138 and EMA were found. Expressions of kappa and lambda light chains confirmed the plasma cell polyclonality, which are the most important factors to distinguish PCG from plasmacytoma. The diagnosis of PCG was eventually confirmed.

PCG is not universally recognized as a distinct entity. Many different names were ever used to describe this lesion, such as plasma cell pseudotumor, inflammatory myofibroblastic tumor (IMT) or pseudotumor and inflammatory fibrosarcoma, of which inflammatory pseudotumor was the most commonly called [5, 13, 14]. The World Health Organization Classification of Tumors of Soft Tissue and Bone regarded PCG as a synonym for IMT [15]. Some even considered it under the heterogeneous group of inflammatory pseudotumor or IMT [3]. Due to the variation in histologic appearance, the term PCG currently represents a group of lesions demonstrating nonspecific chronic inflammatory changes rather than a single entity [2, 16]. The exact etiology of PCG remains unclear, although it is thought to be reactive. Plasma cell infiltration in connective tissue may be observed due to infectious, autoimmune, idiopathic, reactive, and malignant stimulations [17]. The common microorganisms associated with the lesion include mycobacteria, Epstein–Barr virus, actinomycetes, nocardia and mycoplasma [5, 14]. One case study even reported amlodipine-induced PCG of the gingiva, revealing the potential contribution of drug-cellular interaction in the pathogenesis of this entity [18]. Recent studies supported PCG as a part of IgG4-related disease [3, 19–23], which was defined as a combined presence of the increased numbers of IgG4 positive plasma cells and the characteristic histopathological appearance. The histopathological features included a dense lymphoplasmacytic infiltration, a storiform pattern of fibrosis, and an obliterative phlebitis [24]. A confirmed relation of PCG with IgG4 may provide significant clinical significance for the treatment of this disease.

There is no consensus regarding therapeutic approaches for PCG. The available treatments include surgical excision, radiotherapy, administration of immunosuppressors (cyclosporine) and corticotherapy [2, 25–27]. PCG is hard to differentiate from a malignancy and histologic frozen section or fine needle aspiration is often inconclusive [28]. Because of the invasive feature, surgical resection is considered to be the first-line treatment for PCG and could be an effective way to confirm the diagnosis, as the case in the present study. Patients with unresectable PCG usually receive radiotherapy or maintenance therapy with corticosteroids [29]**.** Christian Schneider et al. even recommended rituximab for PCG in cases which are refractory to corticosteroid treatment or inaccessible to complete surgical excision [25]. However, those conservative treatments may have significant side effects while failing to reduce the mass [23, 30]**.** In addition, recurrence is also observed, especially in cases of subtotal excision, which emphasizes the need for more specific long-term therapies with fewer adverse effects [4, 31].

PCG is a rare non-neoplastic lesion characterized by the presence of polyclonal plasma cells. We presented the first case of PCG occurred in the thoracolumbar spine, which was diagnosed eventually by pathological examination of completely excised specimen. Surgical excision is preferred for the treatment of spinal PCG. Cases with surgical contraindication can choose glucocorticoids or radiation therapy. Since the etiology of the present case of PCG remains unclear, more studies are required to identify the mechanism of this disease so as to improve the clinical outcome.

## Data Availability

All data generated or analyzed during this study are included in this published article.

## Abbreviations

PCG: Plasma cell granuloma
CT: Computed tomography
HIV: Human immunodeficiency virus
MR: Magnetic resonance
GM: Galactomannan
MM: Multiple myeloma
T-SPOT: T Cell spot test
RPR: Rapid plasma reagin
HE: Hematoxylin and eosin
CD: Cluster of differentiation
EMA: Epithelial membrane antigen
LCA: Leukocyte common antigen
IMT: Inflammatory myofibroblastic tumor
IgG: Immunoglobulin G
